# Fucoidan–Fucoxanthin Ameliorated Cardiac Function via IRS1/GRB2/ SOS1, GSK3β/CREB Pathways and Metabolic Pathways in Senescent Mice

**DOI:** 10.3390/md17010069

**Published:** 2019-01-21

**Authors:** Po-Ming Chang, Kuan-Lun Li, Yen-Chang Lin

**Affiliations:** Graduate Institute of Biotechnology, Chinese Culture University, Taipei 11114, Taiwan; aming800123@gmail.com (P.-M.C.); noodleplusalan@gmail.com (K.-L.L.)

**Keywords:** fucoidan, fucoxanthin, aging mice, metabolomics, long QT

## Abstract

The effects of low molecular weight fucoidan (LMWF) in combination with high-stability fucoxanthin (HSFUCO) on cardiac function and the metabolic pathways of aging mice (*Mus musculus*) were investigated. We demonstrated that LMWF and HSFUCO could improve cardiac function in aging mice. Aging mice were treated with LMWF and HSFUCO, either on their own or in combination, on 28 consecutive days. Electrocardiography and whole-cell patch-clamp were used to measure QT interval and action potential duration (APD) of the subjects. Cardiac tissue morphology, reactive oxygen species, and Western blot were also applied. Ultra-high-performance liquid chromatography–quadrupole time-of-flight (UPLC-QTOF) mass spectrometry was used for investigating metabolic alterations. The use of LMWF and HSFUCO resulted in improvements in both ventricular rhythms (QT and APD). Treatment with fucoidan and fucoxanthin reduced the expression levels of SOS1 and GRB2 while increasing GSK3β, CREB and IRS1 proteins expression in the aging process. Three main metabolic pathways, namely the TCA cycle, glycolysis, and steroid hormone biosynthesis, were highly enriched in the pathway enrichment analysis. When taken together, the LMWF and HSFUCO treatment improved both the ventricular rhythm and the muscular function of aging subjects by interfering with the metabolism and gene function.

## 1. Introduction

Aging is a process accompanied by many associated degenerative diseases, such as cancer, cardiovascular disease, and dementia [1]. Cardiac structural remodeling and muscular atrophy are the most severe forms of cardiac disease [2,3,4]. There are many approaches to curing cardiovascular disease and, recently, natural products derived from herbs have been extensively studied for their ability to improve cardiovascular function [5,6,7]. Numerous brown seaweeds contain this sulfated, fucosylated polysaccharide phytochemical compound. The biological and chemical properties of fucoidan vary according to the particular type of brown seaweed and the extraction method employed [8]. Antitumor [9], antiviral [10], anti-inflammatory [11], and anticoagulant [12] activities are the main properties of this compound, which are strongly associated with its molecular weight [13] and sulfate content [14]. Fucoidan has long been known as a good antioxidant and anti-inflammatory compound [15,16,17,18,19]. Recently, several studies have focused on low molecular weight fucoidan due to its higher level of biological activity [15,20,21,22,23]. Fucoidan has also been used with myocardial infarction in a rat model, and here it showed a cardioprotective effect by reversing the damage to the region induced by isoproterenol [24]. Fucoidan can protect against the damage caused by myocardial ischemia–reperfusion [25]. Low molecular weight fucoidan (LMWF) possesses higher pharmacological properties than high molecular weight (HMWF) fucoidan [26].

Fucoxanthin, a carotenoid compound, is found in the chloroplasts of brown seaweed. This phytochemical has highly anti-inflammatory and antioxidant properties [27,28]. Many studies have been conducted that show the cardioprotective effect of fucoidan [24,29]. However, no study has used a combination of LMWF and high-stability fucoxanthin (HSFUCO) to treat aging-related cardiac disease. 

Glycogen synthase kinase-3 (GSK-3) is a crucial regulator of cardiac hypertrophy, and 2,5-dimethylcelecoxib (DM-celecoxib) was found to activate Grb2 tyrosine phosphorylated during the aging process stimulated by CD4. This tyrosine phosphorylation may activate through signals from the *Ras* pathway from T cell [30]. In human subjects, the c-Src/Pyk2/EGFR/CREB-dependent pathway is known to be involved in cardiac hypertrophy [31,32]. Hutter et al. found out that the decline in Ras/ERK mitogen-activated protein kinase cascade was related to aging. One highly homologous serine/threonine protein kinase called p90RSK may phosphorylate transcription factors, including c-fos, CREB, and IκB [33]. In addition, She-GRB2-SOS is also known to be an important pathway in the cardiac myocytes [34].

IRS-1 is known for its role in the regulation of cell and body size, and it is one of membranous or cytoplasmic proteins [35]. In addition, one former study related to lifespan and aging indicated Irs1^−/−^ mice are long-living [36]. 

Taking advantage of publicly available transcriptomics database, we aim to mine for potent gene signal pathways related to LMW fucoidan and high-stability fucoxanthin treatment and cardiac function on aging mice subjects. Furthermore, the metabolomics approach was used to identify secondary metabolites as novel biomarkers to distinguish between young and aging mice with and without LMW fucoidan and high-stability fucoxanthin treatment. 

## 2. Materials and Methods 

### 2.1. Animals and LMWF/HSFUCO Administration

Eight-week-old and two-year-old male C57BL/6 mice were purchased from the National Laboratory Animal Center. The animals were raised under standard laboratory conditions with a 12 h light/12 h dark cycle and food and water ad libitum.

There were six mice in each of the five experimental groups. The first group (1) was a young control group (eight-week-old male mice) (YC group). In the other four groups, two-year-old male C57BL/6 mice were used. These groups were (2) the aging control group (SC group), (3) aging mice treated with fucoidan (Hi-Q Oligo-Fucoidans^®^) (500 mg/kg) (FD group), (4) aging mice treated with HS fucoxanthin (HSFUCO) (500 mg/kg) (FX group), (5) aging mice treated with fucoidan (250 mg/kg) plus HSFUCO (250 mg/kg) (FD + FX group). Hi-Q Oligo-Fucoidans^®^ and HSFUCO were derived from *Sargassum hemiphyllum* and prepared by Hi-Q Marine Biotech International Ltd. (New Taipei City, Taiwan). A sixth, quality control (QC) group was included in the experiments. All treatments with fucoindan and fucoxanthin were fed orally to mice.

At days 1 and 28, we measured forelimb grip strength, exhaustive swimming time, and electrocardiogram (ECG) and action potential (by use of a patch-clamp). At the end of the experiment, the mice were sacrificed and the whole hearts collected for H&E and Masson’s trichrome staining.

All the experiments involving animals were approved by the Institutional Animal Care and Use Committee (IACUC), with approval number CCU-IACUC-105-008 (Approval day: 28th December 2015), Chinese Culture University, Taiwan, ROC. The study period was from 1st August 2016 to 31st July 2017. The experiment complied with the Guide for the Care and Use of Laboratory Animals published by the National Research Council (revised 2011) and the Guide for the Care and Use of Laboratory Animals—Taiwanese Edition (1996). Isoflurane anesthesia was used to reduce the subjects’ suffering.

### 2.2. Grip Strength Test

To evaluate the muscular strength of the mice, the laboratory of Huang Qizhang developed a measuring device for their forelimb gripping strength. Used to measure a mouse’s forelimb grip, it provides an assessment of the effect of drugs, toxins, muscle relaxants, disease, aging, and nerve damage on muscle strength. The test animals were placed on a test bench, and the front of the head were fitted with a force sensor grab bar. The animal will instinctively grasp the grab bar in front of it and resist backward movement until the pull exerted by the experimenter exceeds the maximum grip of the mouse. Analysis of changes in forelimb gripping strength following treatment with fucoidan and fucoxanthin can provide insight into muscle strength enhancement [37]. This experiment was carried out at the National Sports University. Results were collected from 6 mice per group.

### 2.3. Exhaustive Swimming Time Test

The effects of fucoidan and fucoxanthin on muscle endurance were also evaluated by the swimming performance of mice. The mice were placed in a 15 cm diameter, 20 cm depth, glass cylinder at 37 ± 1 °C (the tank diameter and water depth were adjusted visually according to mouse size), and the mice were forced to swim until they were exhausted. This was taken as the point when the body of the mouse, including its head, was under the water for 8 seconds without being able to surface [37]. This experiment was carried out at the National Sports University. The results were collected from 6 mice per group.

### 2.4. Reactive Oxygen Species and Aging

Blood samples were collected while the rats were sacrificed. A 1 ml blood sample was placed in a collection tube (containing Heparin). The upper plasma and buffy coat were removed by centrifugation (1500–2000 rpm/5–10 min). The blood cells were washed by adding 2 mL of PBS, centrifuged again at 1000 rcf for 5 min) and the supernatant was discarded. The above steps were repeated three times.

We added Dulbecco’s modification of Eagle medium (DMEM without FBS) in red blood cells and calculated the total cell count. Filuted 100 μL red blood cell suspension was added into a 96-well plate for each (100,000 cells per well) together with 0, 0.5, 1, 5 times concentration of 50 μL AAPH, respectively. Waiting for the 3 h reaction, we then added PBS to wash. After washing, 100 μL of 10 μM H_2_DCF-DA (2,7-dichloridihydrofluorescein, Sigma) were added, then the mixture was incubated for 30 min, washed by PBS, centrifuged at 1000 rcf for 5 min, and then the supernatant was discarded. The above steps were repeated twice. Finally, 100 μL and 180 μL of Triton X-100 were added to break the cells in a black 96-well plate. The absorbance was measured by cytofluormetry. The excitation wavelength was set to 485 nm and the emission wavelength was set at 527 nm. According to the analysis results, the lower the devaluation was, the lower ROS concentration in blood.

### 2.5. Measurements from Electrocardiogram (ECG)

The ECG of the mice was measured using a three leads vector connected to a PowerLab/4SP analog-to-digital converter, from AD Instruments, Colorado Springs, CO. Isoflurane 2% mixed with compressed oxygen at 2 liters/min flow rate was used for the anesthesia. The heart rate and ECG data, including QT and corrected QT interval, were recorded from 6 mice per group.

### 2.6. Isolation of Cardiomyocytes

The mice were anesthetized using sodium pentobarbital (50 mg/kg); the hearts were removed quickly and immersed in a normal Tyrode solution containing heparin. The hearts were then perfused with oxygenated Ca^2+^ free Tyrode solution at 35–37 °C for 10 min, followed by oxygenated Ca^2+^ free Tyrode solution containing 0.6 mg/mL collagenase II (Worthington) for 25–30 min. The hearts were then left in an oxygenated Ca^2+^ free Tyrode solution for 15 min. The ventricles were minced into small pieces in a Kraft-Brühe (KB) solution (mM: taurine 10; glutamic acid 70; KCl 25; KH_2_PO_4_ 10; glucose 22; EGTA 0.5; pH adjusted to 7.2 with KOH), filtered through a 200 µm mesh, and dispersed by gentle shaking.

### 2.7. Urine Sample Preparation

Urine samples were centrifuged at 9000–10,000 rpm, at 4 °C for 5 min. The suspension was collected and MeOH (1:1) was added, following which it was put on ice for a couple of minutes. It was centrifuged again at 9000–10,000 rpm, at 4 °C for 5 min. The suspension was collected and filtered through a 0.45 µm filter. Samples from the five experimental groups (YC, SC, FD, FX, and FD + FX), and from the quality control (QC) group were collected and stored separately. All the processes were conducted on ice.

### 2.8. Metabolomics Analysis 

We started the metabolomics analysis using the ultra-high performance liquid chromatography-quadrupole time-of-flight mass spectrometry (UPLC/Q-TOF MS) system to investigate the changes in secondary metabolites in the aging model and attempted to establish a high throughput biomarkers screening system. We chose urine samples (collected in a non-invasive way) to analyze the secondary metabolites. We used EZ-info software to generate the S-plot figure to investigate the relationships between the 6 different groups.

MetaboAnalyst version 3.0 (http://www.metaboanalyst.ca) was used for the chemometric partial least square-discriminant analysis (PLS-DA) in both 2D and 3D views, and for variable importance in projection (VIP) scores for the metabolites, for pathway analysis, and for the enrichment analysis of the integrated pathway.

### 2.9. Action Potential Measurement in C57BL/6 Mice Myocytes by Patch-Clamp Technique

The method was described previously [38]. In brief, the action potential from the isolated ventricular cardiomyocytes from mice was recorded in Tyrode and pipette solutions. The patch-clamp setup was done with bright-field and fluorescent light sources, and a CCD camera.

### 2.10. Histology (Hematoxylin and Eosin (H&E) Staining)

After the ECG recording was taken, the heart was quickly removed, washed twice in PBS solution, and immersed in 4% paraformaldehyde (PFA) overnight at room temperature. Next, the PFA solution was replaced with 70% ethanol, then fixed in paraffin and cut at 2.5 mm. Afterward, rehydration and staining with hematoxylin and eosin (H&E) were performed.

### 2.11. Masson Trichrome Staining

Masson trichrome staining was done as described in our previous publication [39]. Briefly, alcohol (100% alcohol, 95% alcohol, 70% alcohol) was used to de-paraffinize in stages and rehydrate the cardiac tissue. Bouin’s solution was used for 15 min at 56 °C to improve staining quality. Samples were rinsed for 5–10 min under tap water to remove the yellow color of the picric acid (Bouin’s solution). Next, they were stained in Weigert’s iron hematoxylin for 10 min and washed with PBS buffer for 5 min. The tissues were then stained in Biebrich scarlet–acid fuchsin solution for 5 min and washed in distilled water. The sections then underwent differentiation and dehydration in 75% and 90% alcohol a few times, followed by rinsing in tap water. Finally, the sections were cleared in xylene and mounted with mounting medium.

### 2.12. Western Blot Assay

The Western blot assay was done as described previously [39]. Briefly, the cardiac tissue was isolated and all fat and connective tissue removed. The cardiac tissues were then homogenized with lysis and extraction buffer (Thermo Fisher Scientific Inc.) on ice. Centrifugation was used to remove the large tissue and nuclear fragments at 7000 *g*, 4 °C for 10 min and the supernatant was collected. The protein concentration was measured by Nanovue Plus™ Spectrophotometer (Harvard Bioscience, Inc., October Hill Rd Holliston, MA, USA). Next, 40 µg of the total protein from each sample was loaded onto 10% SDS gel and run for 1 h at 100 mA. Afterward, a polyvinylidine fluoride (PVDF) membrane was used to transfer the protein bands on ice in 1 h at 100 mA. Blocking of the membrane was done with 10% nonfat dry milk (Bio-rad) and then incubated with primary antibodies in 5% milk overnight. The primary antibodies were as follows: Polyclonal antibodies against Phospho-GRB2 antibody [Y237] (ab32037) (1:1000, ABCAM), Phospho- IRS1 [y612] (ab66153) (1:1000, ABCAM), Phospho-SOS1 (ab64595) (1:1000, ABCAM), phosphor-GSK3 beta [Y216] (ab75745) (1:1000, ABCAM), phosphor-CREB [E113] (ab32096) (1:1000, ABCAM), and Actin (1:1000, ABCAM). Afterward, secondary antibodies, with horseradish peroxidase (HRP) (1:10000), were incubated with the membrane at room temperature. The membrane was finally read by a C-DiGit^®^ Blot Scanner (LI-COR Biosciences, New Rochelle, NY, USA).

### 2.13. Statistical Analysis

T-test was used for comparison between control and treated groups. *p*-value < 0.05 was considered as significant difference between groups. Data were displayed as mean ± SD.

### 2.14. Bioinformatics Analysis

We analyzed the GSE76374 dataset, a dataset available to the public at GEO (NCBI) [40] to screen for target genes possibly involved in this process. The DNA microarray raw data were curated and standardized using the Genepix Pro 6.0 software (Molecular Devices). By adjusting to the background value, the corrected intensity was acquired with only a gene expression larger than 2-fold relative to the control sample. The detailed calculation was previously described by Ito et al. [41]. The change and difference in gene expression of the fucoidan group (*n* = 6) relative to the control group (*n* = 6) was computed. Then, Metacore (GeneGo, Inc., 500 Renaissance Dr., Ste. 106, St. Joseph, MI, USA) was applied to perform a search for significant pathways and networks using the input gene set, gene ontology molecular functions, and GeneGo maps (pathways). Finally, the input data (gene with fold change >2) were processed with Metacore software. *p* value < 0.05 was considered statistically significant.

## 3. Results

### 3.1. H&E Staining and Masson’s Trichrome Staining

**Figure 1:**
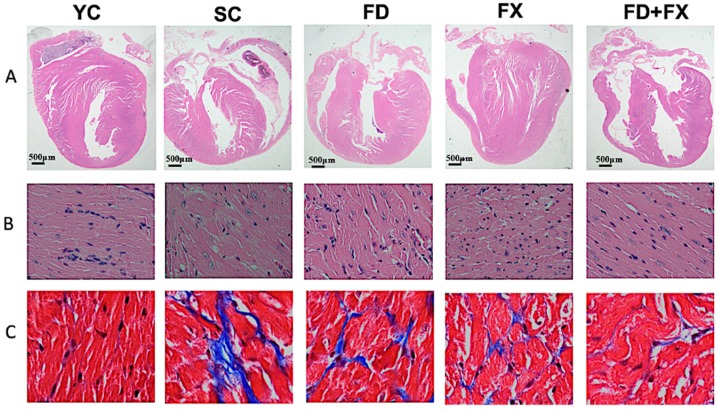
Characterization of the histopathology of the left ventricular tissue in the young mouse control group (YC) and in aging mice treated with fucoidan and fucoxanthin. (**A**) The upper panel displays images of whole hearts stained with hematoxylin and eosin (for observation of ventricular hypertrophy and dilatation). (**B**) An enlarged scale of the upper panel (600×). (**C**) Masson’s trichrome staining of serial ventricle tissue sections. SC: Senescent mice; FD: Senescent mice treated with fucoidan; FX: Senescent mice treated with fucoxanthin; FD + FX: Senescent mice treated with both fucoidan and fucoxanthin. (*n* = 6).

The cardioprotective effects of LMF and HSFUCO were assessed in C57BL/6 mice based on H&E and Masson’s trichrome staining. H&E staining showed cardiac structure alteration in four groups (Figure 1A). The Masson’s trichrome staining showed significant change in the left ventricle of mice treated with LMWF and HSFUCO relative to the YC group. In the YC group, the cardiac muscles were well aligned with less interstitial tissue, while the cardiac tissue from the SC group indicated a high proportion of fibrosis over almost the entire area. In the treated groups, the cardiac fibrosis was significantly reduced in the ventricle areas of the FD, FX, and FD + FX groups (Figure 1B). In summary, the findings from histological examination may considerably clarify malfunctions of the cardiac system, such as the QT interval and action potential alterations, via structural changes in aging mice. These changes could be dramatically reversed to the almost normal conditions when treated with LMWF and HSFUCO, whether in combination or by treating with each compound alone.

### 3.2. Grip Strength and Swimming Exhaustion Test Showed Significant Differences between Aging Mice and Young Controls

**Figure 2:**
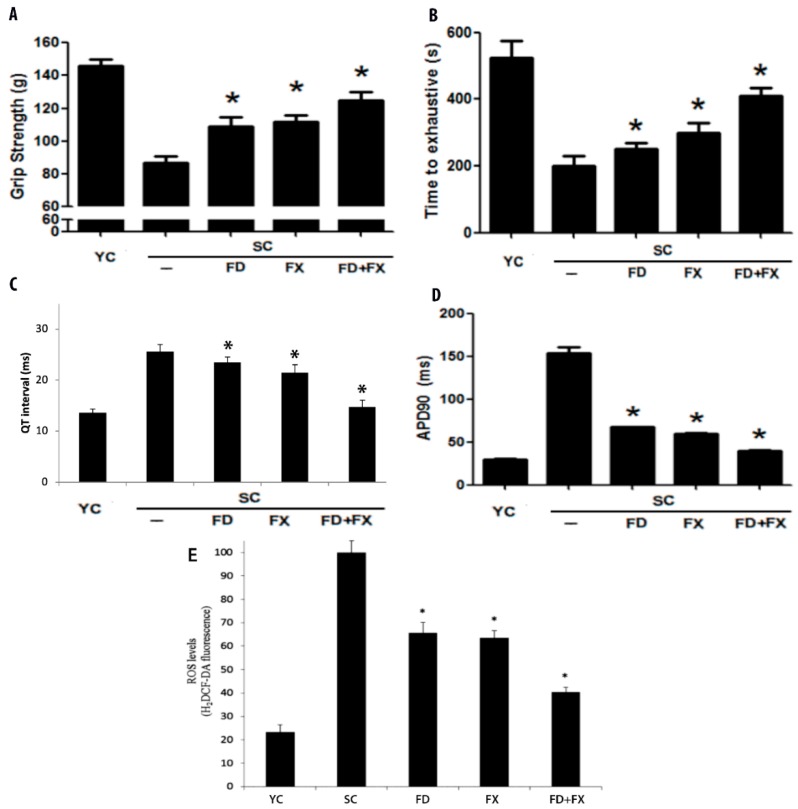
(**A**) Grip strength: Test of forelimb gripping performance (explosive force) by grip strength meter. (**B**) Swimming exhaustion test: Muscle endurance was measured for all groups. (**C**) QT interval was measured in young control, aging, and aging mice fed with fucoidan or/and high-stability fucoxanthin HSFUCO groups. (**D**) Action potential (AP) measurement. (**E**) Reactive oxygen species of 5 groups, including young control, aging, and aging mice fed with fucoidan or/and HSFUCO, are displayed (*n* = 6).

There were significant improvements after treatment with fucoidan and fucoxanthin. The SC group did significantly worse in the forelimb gripping test than the YC group. It is assumed that this was due to decreased muscle function in the aging mice (Figure 2A).

The exhaustive swimming time performance test results are shown in Figure 2B. The aging control group had a significantly lower swimming duration (lower muscle endurance) compared to the YC group. There were also significant differences between the fucoidan and fucoxanthin groups and the YC group (*p* < 0.05). All the aging mice did significantly worse in the swim exhaustion test than the young control group.

### 3.3. Effect of LMF and HSFUCO on QT Interval and Action Potential in Aging Mice Model

Prolonged QT interval was determined in the aging mice control group (SC) (25.6 ± 1.35 msec) relative to the young control group (13.62 ± 0.68 msec). The experimental groups’ QT interval was reduced when treated with LMF and HSFUCO either alone or in combination (FD: 23.42 ± 1.1; FX: 21.39 ± 1.6; FD + FX: 14.71 ± 1.4 msec) (Figure 2C).

Consistent with the results of the prolonged QT interval in the aging mice model, the action potential duration in the myocytes of mice treated with LMWF and HSFUCO showed significant improvement relative to the aging group. The plateau phase was 29.28 ± 1.18 ms in the young control, 153.7 ± 6.47 ms in the aging control, 67.3 ± 0.70 ms in the fucoidan-fed, 59.31 ± 0.96 ms in the fucoxanthin-fed, and 39.84 ± 1.06 ms in mice fed with both fucoidan and fucoxanthin. The plateau phase in the aging control mice was highly extended. Based on our results, the plateau phase extension would be reduced by fucoidan and fucoxanthin. Moreover, the effects of LMWF and HSFUCO were evident in the reduction of QT prolongation and plateau phase extension (Figure 2D). The alteration in action potential found in the ventricular myocytes is possibly due to ionic mechanisms. Among the five groups, the FD + FX group showed the greatest recovery of the QT interval relative to the other treated groups.

### 3.4. Reactive Oxygen Species and Aging

Figure 2E shows a comparison of the five groups, including the young mice control group, the aging mice control group, and the experimental groups of aging mice fed with fucoidan and/or fucoidan continuously for 28 days (*p* < 0.05). A comparison of the young and aging mice shows that the activity of the YC, SC, FD, FX, and FD + FX groups were 23.39%, 100%, 65.58%, 63.57% and 40.34%, respectively. It was found that the reactive oxygen species in the aging mice were considerably higher than those in the young control group. Fucoidan and fucoxanthin treatments resulted in significant improvement relative to the aging control group.

### 3.5. “Development_PIP3 Signaling in Cardiac Myocytes” Pathways are Significantly Modulated during Fucoidan Treatment

**Figure 3:**
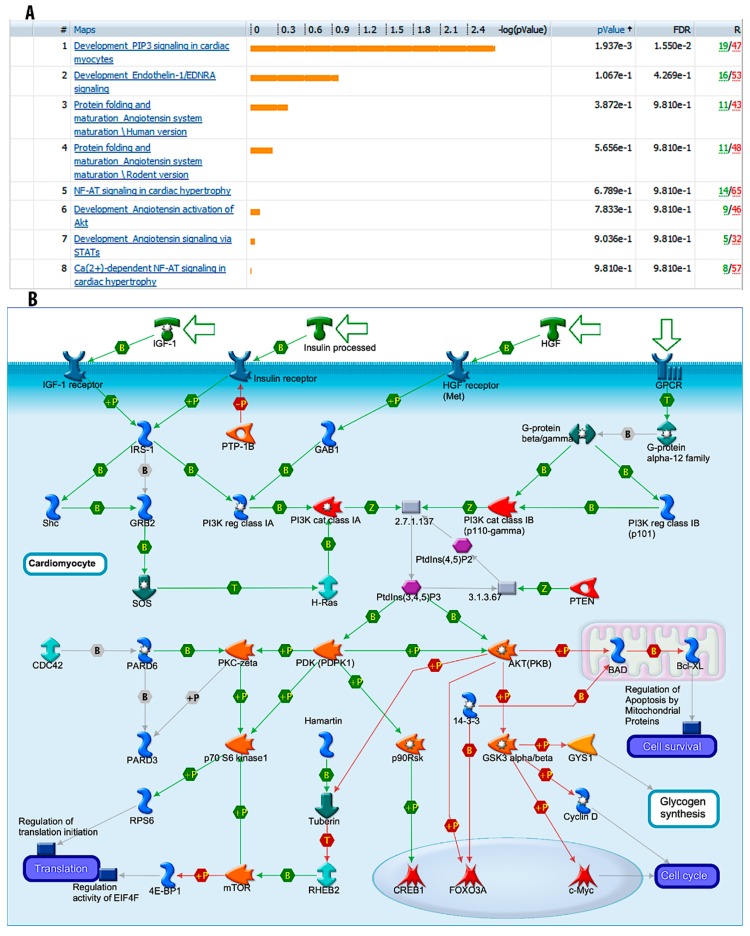
Metacore analysis of significant pathways in aging mice. (**A**) Top pathways involved in the cardiac function of aging mice. (**B**) Detail of “Development_PIP3 signaling in cardiac myocytes” pathway. (*n* = 6).

Using Metacore GeneGo software for gene mapping, it was shown that “Development_PIP3 signaling in cardiac myocytes” was the most significant pathway between the fucoidan-treated group and the control group with *p* value = 5.436 × 10^−13^ and FDR = 2.549 × 10^−12^. This result was derived from 13,175 significant genes with fold change >1.5 (Figure 3A,B). However, the underlying mechanism of fucoidan’s involvement in ameliorating cardiac malfunction is still under investigation.

### 3.6. SOS1, GSK3β, GRB2, CREB, and IRS1 Protein Expression Level in Aging were Ameliorated with Fucoidan and Fucoxanthin

**Figure 4:**
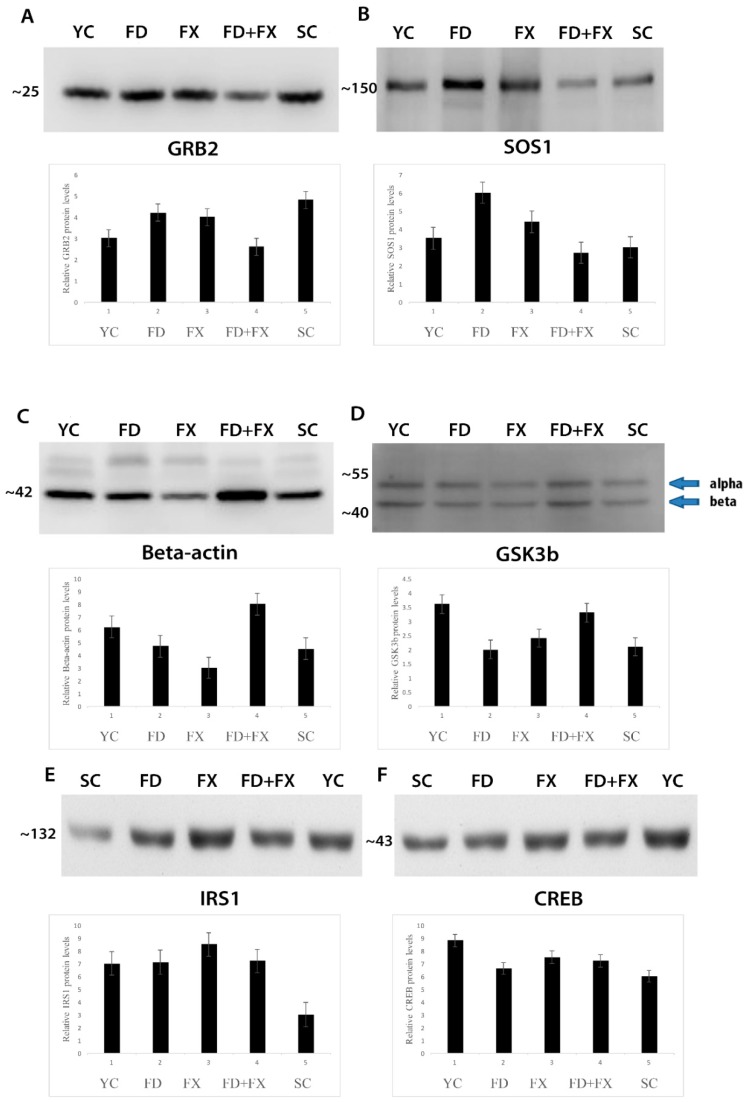
Protein expression level of GRB2 (**A**), SOS1 (**B**), beta-actin (**C**), GSK3β (**D**), IRS1 (**E**) and CREB (**F**), under treatment by fucoidan and fucoxanthin. (*n* = 6).

GRB2 protein expression level was high in the YC, SC, FX, and FD groups but not in the FD + FX group (Figure 4A). SOS1 protein expression level was highest in the FD group. Under treatment by HSFUCO, the expression level of SOS1 was increased compared to that of the aging group. Interestingly, the combined effect of fucoidan and HSFUCO showed a comparable level of SOS1 protein expression to that of the aging group (Figure 4B). The beta-actin levels were not stable in all groups (Figure 4C). The GSK3β protein expression level was higher in the young control subjects than in the aging control group. Fucoidan alone kept the expression level of this protein unchanged, whereas fucoidan in combination with HSFUCO (FD + FX) and HSFUCO (FX) alone showed increases in the level of GSK3β protein (Figure 4D). The IRS1 protein level was also high in YC, FX, FD, and FX + FD but not in the SC group (Figure 4E). The CREB protein was highest in the YC group when compared to the rest of the groups (Figure 4F).

### 3.7. Chemometric Analysis and PLS-DA Showed Positive Effects for Fucoidan and Fucoxanthin

**Figure 5:**
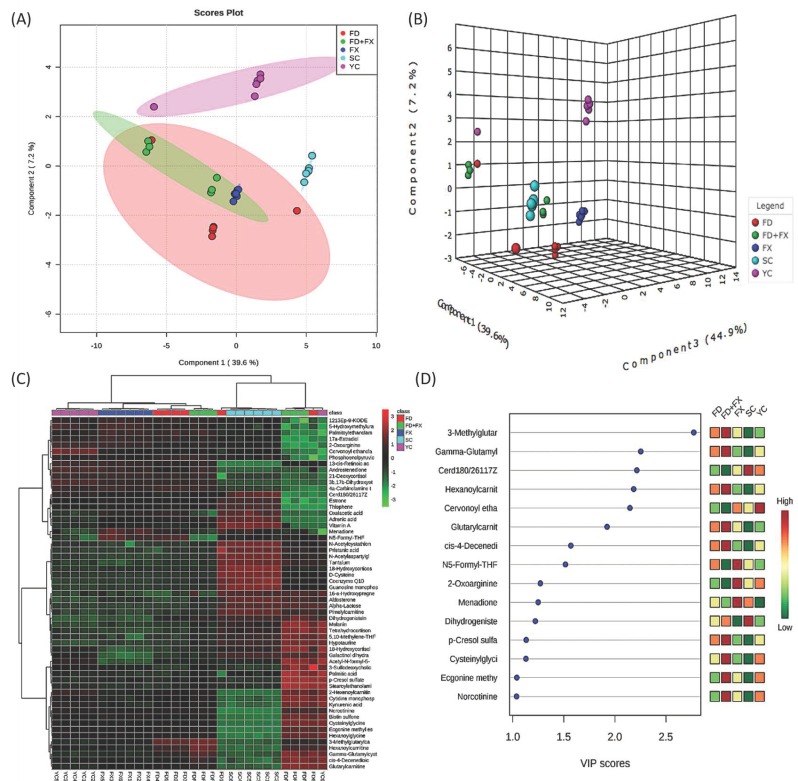
Cluster analysis and partial least square-discriminant analysis (PLS-DA) of five experimental groups. PLS-DA of young mice, aging mice, aging mice treated with fucoidan, aging mice treated with fucoxanthin, and aging mice treated with fucoidan and fucoxanthin. The results of the combination treatment are illustrated in 2D (**A**) and 3D (**B**). (**C**) Heatmap of 57 metabolites is displayed. (**D**) Variable importance in projection (VIP) score of top 15 metabolites in five groups investigated. (*n* = 6).

Chemometric analysis and PLS-DA were used to give a general view of the differences in the metabolites’ expression patterns between the five experimental groups. Basically, the five groups were clearly separated by the expression of metabolites in the 2D and 3D displays (Figure 5A,B). The young control and aging control subjects could be distinguished from the aging subjects treated with fucoidan (FD); however, the FD mice did not come close to the YC group. A heatmap of 57 metabolites is displayed in Figure 5C. The top 15 metabolites with a VIP score >1 for discriminating between these three groups are displayed in Figure 5D.

**Figure 6:**
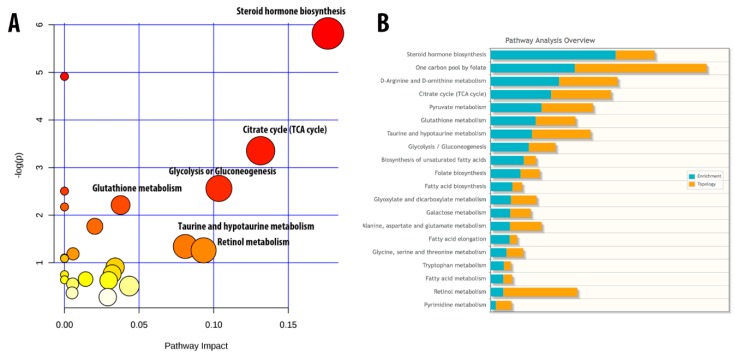
Critical metabolic pathways and integrated pathway involved in the aging process. (**A**) Fifty-seven metabolites involved in multiple metabolic pathways. (**B**) Integrated pathway of 57 metabolites and 5 genes, namely GRB2, SOS1, IRS1, CREB, and GSK3β (*n* = 6).

Steroid hormone biosynthesis, the citrate cycle (TCA cycle), and the glycolysis cycle were the three most significant metabolic pathways involved in the aging process (Figure 6A). An integrated pathway analysis of 57 metabolites and SOS1, GSK3b, GRB2, CREB and IRS1 is displayed in Figure 6B. A list of 57 metabolites has been provided in Supplementary Table S1. A list of pathways has been provided in Supplementary Table S2.

## 4. Discussion

**Figure 7:**
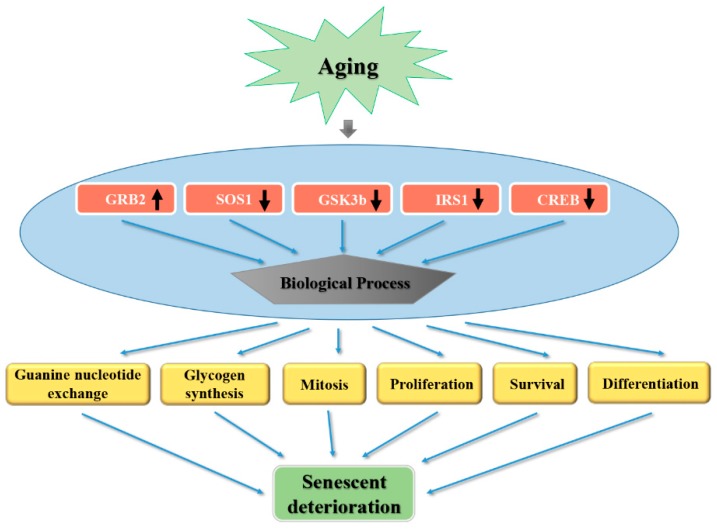
Proposed scheme of aging, showing related proteins and cellular activities.

In the present study, the cardioprotective roles of fucoidan and fucoxanthin were demonstrated through various investigations. Fucoidan and fucoxanthin in combination can potentially reduce cardiac hypertrophy, cardiac fibrosis, ROS level, and shortened QT interval in aging mice subjects. Further investigations into the underlying mechanisms of these phytochemicals extracted from brown algae were also conducted. The data suggest that aging subjects develop cardiac abnormalities due to metabolic disorders in numerous metabolites as well as alterations in gene expressions, such as SOS1, CREB, IRS1, GRB2 and GSK3β. The integrated signal pathways between these genes and the metabolites affected were enhanced, and potential pathways were figured out, such as steroid hormone biosynthesis, one carbon pool by folate, or the TCA cycle. A proposed scheme for the aging process and its related proteins as well as the multiple cellular processes likely to be involved in senescent deterioration is depicted in Figure 7.

QT interval prolongation was demonstrated to have an interdependence with carotid intima-media thickness [42], which in turn generates atherosclerosis [43]. QT interval is a reliable predictor of ventricular arrhythmias [44]. Moreover, QT prolongation associated with cardiac hypertrophy could lead to cardiac sudden death [45]. In the present findings, we found that both ventricular hypertrophy and QT prolongation presented in the aging mice model. The process of membrane depolarization and repolarization (plateau) controls the efficiency of the myocardial or muscle cell contraction and relaxation. Thus, abnormal pacing or conduction can lead to arrhythmias, such as long QT syndromes (LQTS), a precursor to cardiac arrest.

HSFUCO has also drawn attention as an antioxidant phytochemical [46,47,48]. It has also been used to treat obesity and diabetes [49]. There is currently no study using HSFUCO to treat cardiac-related diseases, especially as related to aging. Our study is the first to combine low molecular weight fucoidan with HSFUCO to examine their cardioprotective effects. Our findings demonstrated that the combination of these two phytochemical compounds can enhance the cardiac status of aging mice. They have the potential to reverse QT interval prolongation, action potential, cardiac hypertrophy, and cardiac fibrosis decline.

Active oxide radicals are by-products of normal oxygen metabolism and are involved in cell signaling and in maintaining body homeostasis. However, over time and due to the impact of the external environment, such as exposure to ultraviolet light or heat, etc., the active oxide increases dramatically. These active substances in the cells do great harm, and reactive oxygen species will destroy the cell membrane’s unsaturated fatty acids, producing lipid peroxidation. The reactive oxygen species and lipid peroxidation will then attack the body’s protein and produce an oxidation-related modification reaction [50]. The study also found that, with age, the proportion of oxidative modification of the organs’ proteins increases [51,52]. Protein oxidation products, such as carbonylated proteins and DNA damage products 8-hydroxy-20-deoxyguanosine (8OHdG), serve as biological indicators of aging [53]. Studies have shown that antioxidant capacity is related to sulfate content, and the more sulfate it contains, the more effective it is at removing superoxide radicals [54].

A previous study has shown that blockage of NF-kB can improve myocardial hypertrophy and cardiac function after myocardial infarction [55,56]. Moreover, an angiotensin-converting-enzyme (ACE) inhibitor and angiotensin receptor blocker can also improve cardiac function in heart failure in the rat model [57]. Glycogen synthase kinase-3 (GSK-3) is a crucial regulator of cardiac hypertrophy. In a previous study, 2,5-dimethylcelecoxib (DM-celecoxib) was found to activate GSK-3α and β by inhibiting Akt, and to prevent left ventricular hypertrophy and fibrosis [58]. GRB2 is known to be involved in cardiac hypertrophy and in fibrosis [59]. According to previous study, Grb2 tyrosine phosphorylated during the aging process, stimulated by CD4. This tyrosine phosphorylation may be activated through signals from the Ras pathway from T cell [30]. In human subjects, the c-Src/Pyk2/EGFR/CREB-dependent pathway is known to be involved in cardiac hypertrophy [31,32]. Hutter et al. found out that the decline in the Ras/ERK mitogen-activated protein kinase cascade was related to aging. One highly homologous serine/threonine protein kinase called p90RSK may phosphorylate transcription factors, including c-fos, CREB, and IκB [33]. Compared to our results, CREB protein expression level decreased in the aging group. The lower down expression level may be caused by CREB phosphorylation. Another research focusing on advanced glycation product receptors (AGERs) relating to diabetes and aging mentioned that tyrosine phosphorylation has been shown to cause MAPK activation through Grb2 and Sos. In addition, Sos protein is one guanine nucleotide-exchange factor, resulting in Shc/Grb2 complex formation [60]. Based on this research result, we can confirm that this, consistent with our results, may explain why Grb2 and Sos protein level expressions change drastically with age. IRS-1 is known for its role in the regulation of cell and body size, and it is one of membranous or cytoplasmic proteins [35]. In addition, one former study related to lifespan and aging indicated Irs1^−/−^ mice are long-living [36].

She-GRB2-SOS is also known to be an important pathway in the cardiac myocytes [34]. In our study, the levels of these proteins were changed through supplementing with fucoidan and fucoxanthin. After the treatment, the expression of these proteins in the aging mice came close to those of the young mice subjects.

The citric acid (TCA) cycle plays an important role in various related metabolic pathways. TCA also plays a role in gluconeogenesis, transamination, deamination, and lipogenesis [61]. The precursor of all steroid hormones is cholesterol, mostly provided by plasma lipoproteins circulating as low-density lipoproteins (LDL). Cholesterol participates in corticosteroid biosynthesis [62]. The synthesis and secretion of steroid hormones is closely associated with the progression of aging [63]. A relative increase in intracellular metabolites is evident in senescent cells, including glycolysis, gluconeogenesis, and the pentose–phosphate pathway [64].

## 5. Conclusions

In this study, the protein expression levels of SOS1, GSK3β, GRB2, CREB and IRS1 in aging mice were ameliorated with fucoidan and fucoxanthin. There were also significant improvements in cardiac morphology and muscular function after the aging mice were fed with fucoidan alone or fucoidan supplemented with fucoxanthin. Altogether, these findings indicate the potential for the amelioration of aging through enhancement of the metabolism and gene function of aging subjects using marine products such as fucoidan and fucoxanthin. 

## Supplementary Materials

The following are available online at http://www.mdpi.com/1660-3397/17/1/69/s1, Figure S1: ADP90 trace data; (**a**) YC, (**b**) SC, (**c**) FD, (**d**) FX, (**e**) FD + FX. Figure S2: Representative raw data of ECG measurement. (**a**) Young Mouse, (**b**) Senescence mouse treated with fucoidan, (**c**) Senescence mouse treated with high stability fucoxanthin, (**d**) Senescence mouse treated with fucoidan and high stability fucoxanthin, (**e**) Senescence mouse. Table S1: List of 57 metabolites with full name mentioned in Figure 5C. Table S2: Metabo Analyst result pathways.

## References

[1] Ames B.N., Shigenaga M.K., Hagen T.M. (1993). Oxidants, antioxidants, and the degenerative diseases of aging. Proc. Natl. Acad. Sci. USA.

[2] Jo H.E., Randhawa S., Corte T.J., Moodley Y. (2016). Idiopathic Pulmonary Fibrosis and the Elderly: Diagnosis and Management Considerations. Drugs Aging.

[3] Borlaug B.A. (2016). Cardiac aging and the fountain of youth. Eur. J. Heart Fail..

[4] Santulli G., Iaccarino G. (2016). Adrenergic signaling in heart failure and cardiovascular aging. Maturitas.

[5] Zhang K.J., Zhu J.Z., Bao X.Y., Zheng Q., Zheng G.Q., Wang Y. (2017). Shexiang Baoxin Pills for Coronary Heart Disease in Animal Models: Preclinical Evidence and Promoting Angiogenesis Mechanism. Front. Pharmacol..

[6] Guo M., Liu Y., Shi D.Z. (2016). Cardiovascular Actions and Therapeutic Potential of Tetramethylpyrazine (Active Component Isolated from Rhizoma Chuanxiong): Roles and Mechanisms. Biomed. Res. Int..

[7] Brenyo A., Aktas M.K. (2014). Review of Complementary and Alternative Medical Treatment of Arrhythmias. Am. J. Cardiol..

[8] Fitton J.H. (2011). Therapies from fucoidan; multifunctional marine polymers. Mar. Drugs.

[9] Hsu H.-Y., Lin T.-Y., Hwang P.-A., Chen R.-H., Tsao S.-M., Hsu J. (2012). Fucoidan induces changes in the epithelial-mesenchymal transition and decreases metastasis by enhancing ubiquitin-dependent TGFβ receptor degradation in breast cancer. Carcinogenesis.

[10] Tengdelius M.E., Lee C.-J., Grenegård M., Griffith M., Påhlsson P., Konradsson P. (2014). Synthesis and Biological Evaluation of Fucoidan-Mimetic Glycopolymers through Cyanoxyl-Mediated Free-Radical Polymerization. Biomacromolecules.

[11] Hwang P.-A., Chien S.-Y., Chan Y.-L., Lu M.-K., Wu C.-H., Kong Z.-L., Wu C.-J. (2011). Inhibition of lipopolysaccharide (LPS)-induced inflammatory responses by *Sargassum hemiphyllum* sulfated polysaccharide extract in RAW 264.7 macrophage cells. J. Agric. Food Chem..

[12] Irhimeh M.R., Fitton J.H., Lowenthal R.M. (2009). Pilot clinical study to evaluate the anticoagulant activity of fucoidan. Blood Coagul. Fibrinolysis.

[13] Yang C., Chung D., Shin I.-S., Lee H., Kim J., Lee Y., You S. (2008). Effects of molecular weight and hydrolysis conditions on anticancer activity of fucoidans from sporophyll of *Undaria pinnatifida*. Int. J. Biol. Macromol..

[14] Soeda S., Sakaguchi S., Shimeno H., Nagamatsu A. (1992). Fibrinolytic and anticoagulant activities of highly sulfated fucoidan. Biochem. Pharm..

[15] Wang J., Zhang Q., Zhang Z., Song H., Li P. (2010). Potential antioxidant and anticoagulant capacity of low molecular weight fucoidan fractions extracted from Laminaria japonica. Int. J. Biol. Macromol..

[16] Wang J., Zhang Q., Zhang Z., Li Z. (2008). Antioxidant activity of sulfated polysaccharide fractions extracted from Laminaria japonica. Int. J. Biol. Macromol..

[17] De Souza M.C.R., Marques C.T., Dore C.M.G., da Silva F.R.F., Rocha H.A.O., Leite E.L. (2007). Antioxidant activities of sulfated polysaccharides from brown and red seaweeds. J. Appl. Phycol..

[18] Park H.Y., Han M.H., Park C., Jin C.-Y., Kim G.-Y., Choi I.-W., Kim N.D., Nam T.-J., Kwon T.K., Choi Y.H. (2011). Anti-inflammatory effects of fucoidan through inhibition of NF-κB, MAPK and Akt activation in lipopolysaccharide-induced BV2 microglia cells. Food Chem. Toxicol..

[19] Medeiros V., Queiroz K., Cardoso M., Monteiro G., Oliveira F., Chavante S., Guimaraes L., Rocha H., Leite E. (2008). Sulfated galactofucan from Lobophora variegata: Anticoagulant and anti-inflammatory properties. Biochemistry (Moscow).

[20] Colliec S., Boisson-vidal C., Jozefonvicz J. (1994). A low molecular weight fucoidan fraction from the brown seaweed *Pelvetia canaliculata*. Phytochemistry.

[21] Luyt C.-E., Meddahi-Pellé A., Ho-Tin-Noe B., Colliec-Jouault S., Guezennec J., Louedec L., Prats H., Jacob M.-P., Osborne-Pellegrin M., Letourneur D. (2003). Low-molecular-weight fucoidan promotes therapeutic revascularization in a rat model of critical hindlimb ischemia. J. Pharm. Exp. Ther..

[22] Deux J.-F., Meddahi-Pellé A., Le Blanche A.F., Feldman L.J., Colliec-Jouault S., Brée F., Boudghène F., Michel J.-B., Letourneur D. (2002). Low molecular weight fucoidan prevents neointimal hyperplasia in rabbit iliac artery in-stent restenosis model. Arterioscler. Thromb. Vasc. Biol..

[23] Lake A.C., Vassy R., Di Benedetto M., Lavigne D., Le Visage C., Perret G.Y., Letourneur D. (2006). Low molecular weight fucoidan increases VEGF165-induced endothelial cell migration by enhancing VEGF165 binding to VEGFR-2 and NRP1. J. Biol. Chem..

[24] Thomes P., Rajendran M., Pasanban B., Rengasamy R. (2010). Cardioprotective activity of Cladosiphon okamuranus fucoidan against isoproterenol induced myocardial infarction in rats. Phytomedicine.

[25] Li C., Gao Y., Xing Y., Zhu H., Shen J., Tian J. (2011). Fucoidan, a sulfated polysaccharide from brown algae, against myocardial ischemia–reperfusion injury in rats via regulating the inflammation response. Food Chem. Toxicol..

[26] Park S.B., Chun K.R., Kim J.K., Suk K., Jung Y.M., Lee W.H. (2010). The differential effect of high and low molecular weight fucoidans on the severity of collagen-induced arthritis in mice. Phytother. Res..

[27] Heo S.J., Yoon W.J., Kim K.N., Ahn G.N., Kang S.M., Kang D.H., Affan A., Oh C., Jung W.K., Jeon Y.J. (2010). Evaluation of anti-inflammatory effect of fucoxanthin isolated from brown algae in lipopolysaccharide-stimulated RAW 264.7 macrophages. Food Chem. Toxicol..

[28] Tan C.P., Hou Y.H. (2014). First evidence for the anti-inflammatory activity of fucoxanthin in high-fat-diet-induced obesity in mice and the antioxidant functions in PC12 cells. Inflammation.

[29] Ha S. (2009). Methods of Treatment of Cardiovascular and Cerebrovascular Diseases with Fucoidan. U.S. Patent.

[30] Ghosh J., Miller R.A. (1995). Rapid tyrosine phosphorylation of Grb2 and Shc in T cells exposed to anti-CD3, anti-CD4, and anti-CD45 stimuli: Differential effects of aging. Mech. Ageing Dev..

[31] Chien T.-Y., Yang C.-M. (2015). Human Cardiac Hypertrophy Induced by Thrombin/COX-2 is Mediated Through a c-Src/Pyk2/EGFR/CREB-dependent Pathway. FASEB J..

[32] Chien P.T.-Y., Lin C.-C., Hsiao L.-D., Yang C.-M. (2015). c-Src/Pyk2/EGFR/PI3K/Akt/CREB-activated pathway contributes to human cardiomyocyte hypertrophy: Role of COX-2 induction. Mol. Cell. Endocrinol..

[33] Hutter D., Yo Y., Chen W., Liu P., Holbrook N.J., Roth G.S., Liu Y. (2000). Age-related decline in Ras/ERK mitogen-activated protein kinase cascade is linked to a reduced association between Shc and EGF receptor. J. Gerontol. Ser. A Biol. Sci. Med. Sci..

[34] Sadoshima J.-I., Izumo S. (1996). The heterotrimeric G q protein-coupled angiotensin II receptor activates p21 ras via the tyrosine kinase-Shc-Grb2-Sos pathway in cardiac myocytes. EMBO J..

[35] Sun H., Tu X., Prisco M., Wu A., Casiburi I., Baserga R. (2003). Insulin-like growth factor I receptor signaling and nuclear translocation of insulin receptor substrates 1 and 2. Mol. Endocrinol..

[36] Selman C., Lingard S., Choudhury A.I., Batterham R.L., Claret M., Clements M., Ramadani F., Okkenhaug K., Schuster E., Blanc E. (2008). Evidence for lifespan extension and delayed age-related biomarkers in insulin receptor substrate 1 null mice. FASEB J..

[37] Wu R.-E., Huang W.-C., Liao C.-C., Chang Y.-K., Kan N.-W., Huang C.-C. (2013). Resveratrol protects against physical fatigue and improves exercise performance in mice. Molecules.

[38] Huang J., Huang A., Zhang Q., Lin Y.-C., Yu H.-G. (2008). Novel mechanism for suppression of hyperpolarization-activated cyclic nucleotide-gated pacemaker channels by receptor-like tyrosine phosphatase-α. J. Biol. Chem..

[39] Phan N.N., Wang C.-Y., Lin Y.-C. (2014). The novel regulations of MEF2A, CAMKK2, CALM3, and TNNI3 in ventricular hypertrophy induced by arsenic exposure in rats. Toxicology.

[40] Yokota T., Nomura K., Nagashima M., Kamimura N. (2016). Fucoidan alleviates high-fat diet-induced dyslipidemia and atherosclerosis in ApoE shl mice deficient in apolipoprotein E expression. J. Nutr. Biochem..

[41] Ito T., Asakura K., Tougou K., Fukuda T., Kubota R., Nonen S., Fujio Y., Azuma J. (2007). Regulation of cytochrome P450 2E1 under hypertonic environment through TonEBP in human hepatocytes. Mol. Pharm..

[42] Ohnishi K., Yoshida H., Shigeno K., Nakamura S., Fujisawa S., Naito K., Shinjo K., Fujita Y., Matsui H., Takeshita A. (2000). Prolongation of the QT interval and ventricular tachycardia in patients treated with arsenic trioxide for acute promyelocytic leukemia. Ann. Intern. Med..

[43] Lamm S.H., Engel A., Penn C.A., Chen R., Feinleib M. (2006). Arsenic cancer risk confounder in southwest Taiwan data set. Environ. Health Perspect..

[44] Roden D.M. (2008). Keep the QT interval: It is a reliable predictor of ventricular arrhythmias. Heart Rhythm.

[45] Kang Y.J. (2006). Cardiac hypertrophy: A risk factor for QT-prolongation and cardiac sudden death. Toxicol. Pathol..

[46] Yan X., Chuda Y., Suzuki M., Nagata T. (1999). Fucoxanthin as the major antioxidant in *Hijikia fusiformis*, a common edible seaweed. Biosci. Biotechnol. Biochem..

[47] Fung A., Hamid N., Lu J. (2013). Fucoxanthin content and antioxidant properties of *Undaria pinnatifida*. Food Chem..

[48] Nomura T., Kikuchi M., Kubodera A., Kawakami Y. (1997). Proton-donative antioxidant activity of fucoxanthin with 1,1-diphenyl-2-picrylhydrazyl (DPPH). IUBMB Life.

[49] Maeda H. (2015). Nutraceutical effects of fucoxanthin for obesity and diabetes therapy: A review. J. Oleo Sci..

[50] Bejma J., Ji L.L. (1999). Aging and acute exercise enhance free radical generation in rat skeletal muscle. J. Appl. Physiol..

[51] Petropoulos I., Friguet B. (2005). Protein maintenance in aging and replicative senescence: A role for the peptide methionine sulfoxide reductases. Biochim. Biophys. Acta (BBA)-Proteins Proteom..

[52] Stadtman E.R., Van Remmen H., Richardson A., Wehr N.B., Levine R.L. (2005). Methionine oxidation and aging. Biochim. Biophys. Acta (BBA)-Proteins Proteom..

[53] Voss P., Siems W. (2006). Clinical oxidation parameters of aging. Free Radic. Res..

[54] Kusaykin M., Bakunina I., Sova V., Ermakova S., Kuznetsova T., Besednova N., Zaporozhets T., Zvyagintseva T. (2008). Structure, biological activity, and enzymatic transformation of fucoidans from the brown seaweeds. Biotechnol. J..

[55] Kawamura N., Kubota T., Kawano S., Monden Y., Feldman A.M., Tsutsui H., Takeshita A., Sunagawa K. (2005). Blockade of NF-κB improves cardiac function and survival without affecting inflammation in TNF-α-induced cardiomyopathy. Cardiovasc. Res..

[56] Kawano S., Kubota T., Monden Y., Tsutsumi T., Inoue T., Kawamura N., Tsutsui H., Sunagawa K. (2006). Blockade of NF-κB improves cardiac function and survival after myocardial infarction. Am. J. Physiol.-Heart Circ. Physiol..

[57] Kim S., Yoshiyama M., Izumi Y., Kawano H., Kimoto M., Zhan Y., Iwao H. (2001). Effects of combination of ACE inhibitor and angiotensin receptor blocker on cardiac remodeling, cardiac function, and survival in rat heart failure. Circulation.

[58] Fujita A., Takahashi-Yanaga F., Morimoto S., Yoshihara T., Arioka M., Igawa K., Tomooka K., Hoka S., Sasaguri T. (2017). 2,5-Dimethylcelecoxib prevents pressure-induced left ventricular remodeling through GSK-3 activation. Hypertens. Res..

[59] Zhang S., Weinheimer C., Courtois M., Kovacs A., Zhang C.E., Cheng A.M., Wang Y., Muslin A.J. (2003). The role of the Grb2–p38 MAPK signaling pathway in cardiac hypertrophy and fibrosis. J. Clin. Investig..

[60] Cai W., He J.C., Zhu L., Lu C., Vlassara H. (2006). Advanced glycation end product (AGE) receptor 1 suppresses cell oxidant stress and activation signaling via EGF receptor. Proc. Natl. Acad. Sci. USA.

[61] Akram M. (2014). Citric acid cycle and role of its intermediates in metabolism. Cell Biochem. Biophys..

[62] Gwynne J.T., Strauss J.F. (1982). The role of lipoproteins in steroidogenesis and cholesterol metabolism in steroidogenic glands. Endocr. Rev..

[63] Zaidi S.K., Shen W.-J., Azhar S. (2012). Impact of aging on steroid hormone biosynthesis and secretion. Open Longev. Sci.

[64] James E.L., Michalek R.D., Pitiyage G.N., de Castro A.M., Vignola K.S., Jones J., Mohney R.P., Karoly E.D., Prime S.S., Parkinson E.K. (2015). Senescent human fibroblasts show increased glycolysis and redox homeostasis with extracellular metabolomes that overlap with those of irreparable DNA damage, aging, and disease. J. Proteome Res..

