# Supplementary material for: Fucoidan–Fucoxanthin Ameliorated Cardiac Function via IRS1/GRB2/ SOS1, GSK3β/CREB Pathways and Metabolic Pathways in Senescent Mice
Source: Mar Drugs. 2019 Jan 21;17(1):69. doi: 10.3390/md17010069 (PMC6356397; doi:10.3390/md17010069)
Supplement: Supplementary file 1 [file marinedrugs-17-00069-s001.pdf]

Fucoidan–Fucoxanthin Ameliorated Cardiac Function via IRS1/GRB2/ SOS1, GSK3β/CREB Pathways and Metabolic Pathways in Senescent Mice

Po-Ming Chang, Kuan-Lun Li and Yen-Chang Lin *

Graduate Institute of Biotechnology, Chinese Culture University, Taipei 11114, Taiwan; aming800123@gmail.com (P.-M.C.); noodleplusalan@gmail.com (K.-L.L.)

* Correspondence: lycnthu@gmail.com or lyc10@ulive.pccu.edu.t; Tel.: 02-28610511 ext31832; Fax: 02-2861-8266.

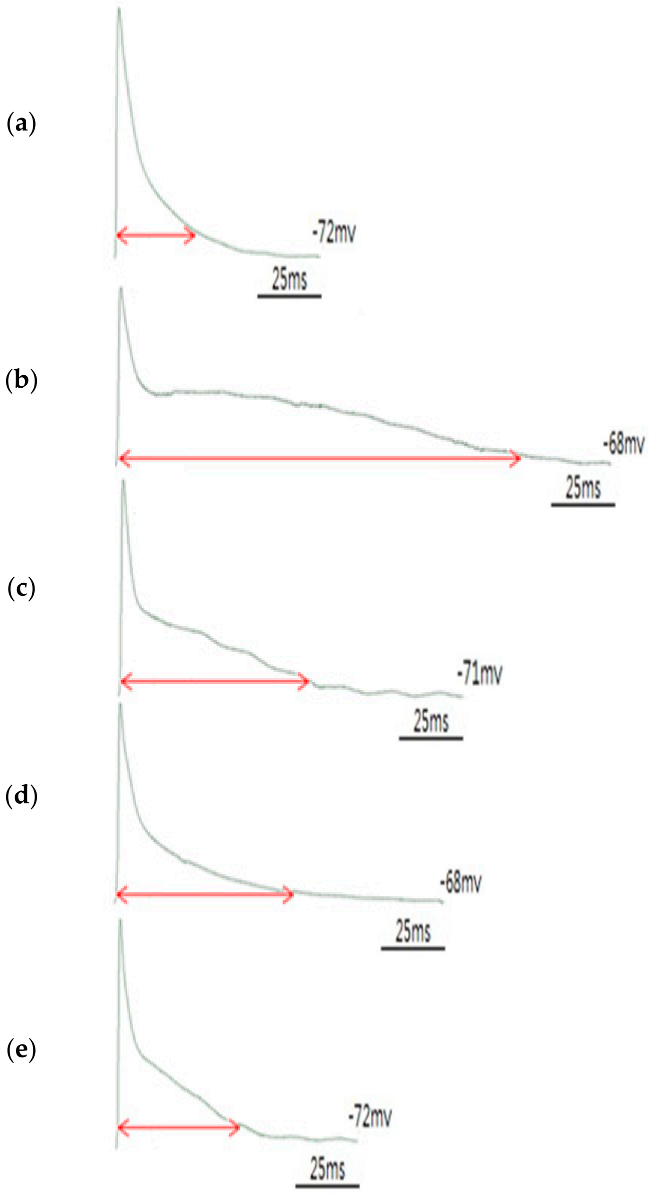

Figure S1. ADP90 trace data; (**a**) YC, (**b**) SC, (**c**) FD, (**d**) FX, (**e**) FD + FX.

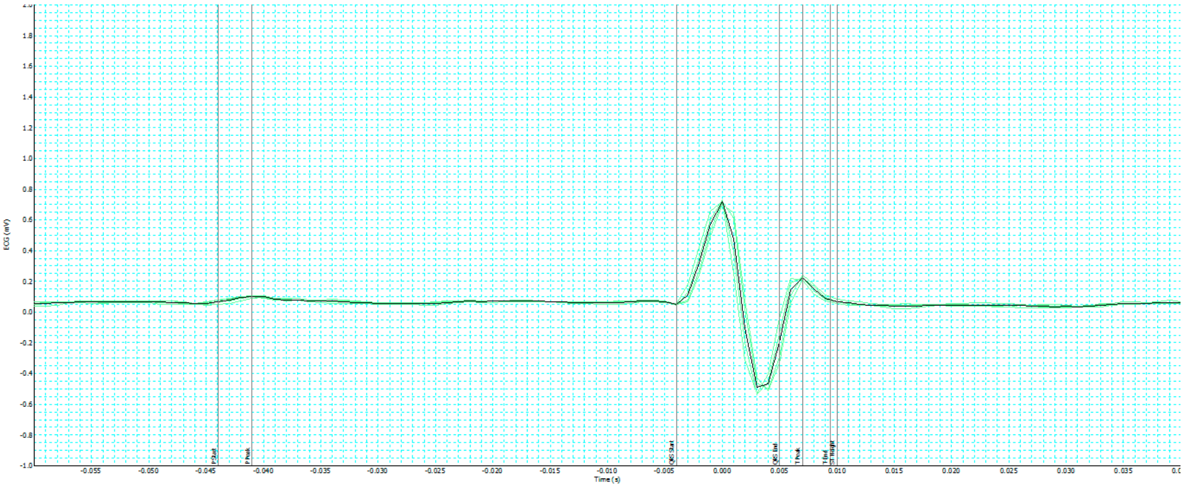

(**a**)

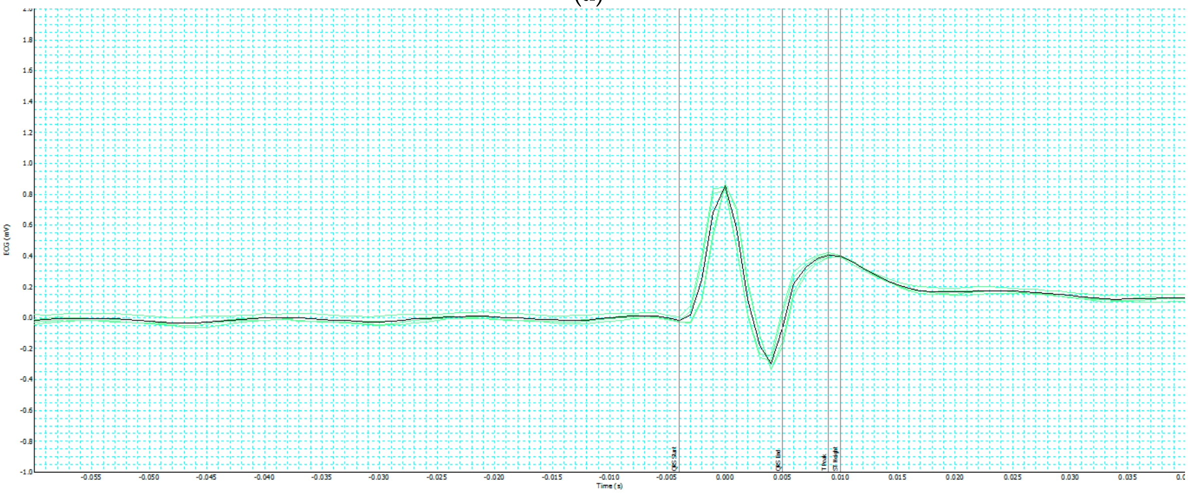

(**b**)

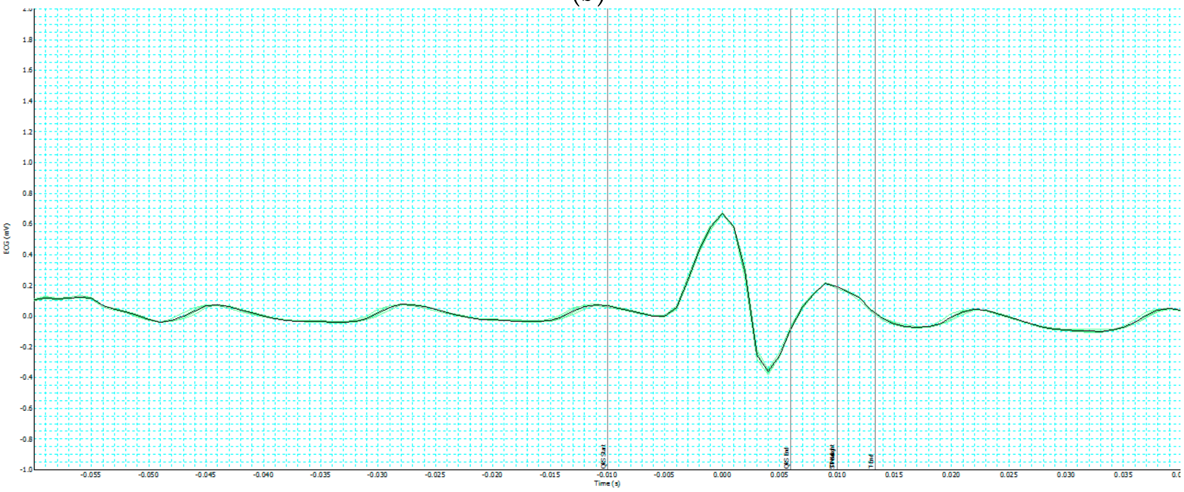

(**c**)

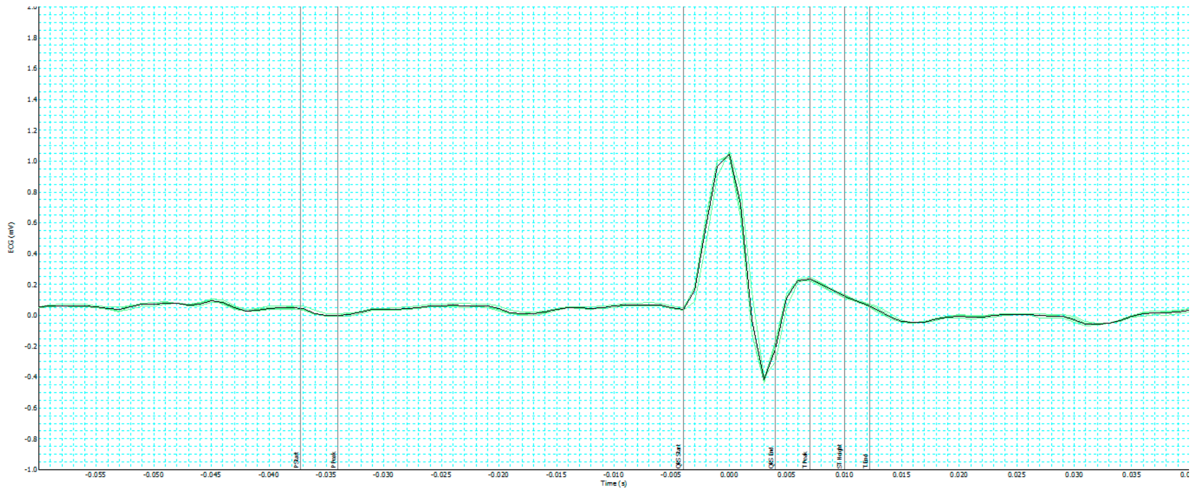

(**d**)

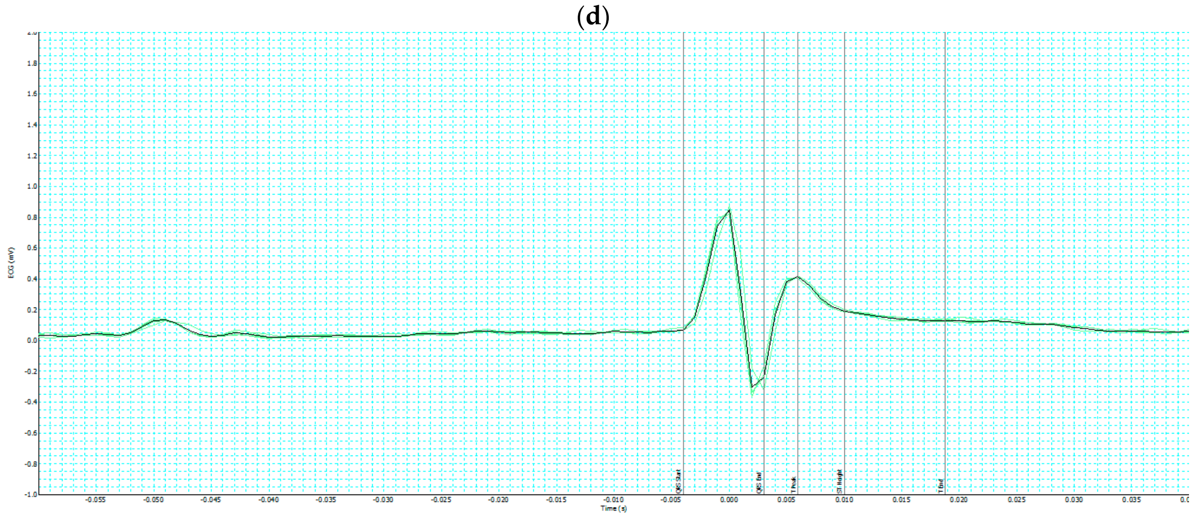

(**e**)

Figure S2. Representative raw data of ECG measurement. (**a**) Young Mouse, (**b**) Senescence mouse treated with fucoidan, (**c**) Senescence mouse treated with high stability fucoxanthin, (**d**) Senescence mouse treated with fucoidan and high stability fucoxanthin, (**e**) Senescence mouse.

Table S1. List of 57 metabolites with full name mentioned in Figure 5C.

No.	Chemical Name	No.	Chemical Name	No.	Chemical Name
1	12(13)Ep-9-KODE	20	Androstenedione	39	Kynurenic acid
2	13-cis-Retinoic acid	21	Biotin sulfone	40	Melanin
3	16-a-Hydroxypregnenolone	22	Cer(d18:0/26:1(17Z))	41	Menadione
4	17a-Estradiol	23	Cervonoyl ethanolamide	42	N-Acetylaspartylglutamic acid
5	18-Hydroxycorticosterone	24	cis-4-Decenedioic acid	43	N-Acetylcystathionine
6	18-Hydroxycortisol	25	Coenzyme Q10	44	N5-Formyl-THF
7	2-Hexenoylcarnitine	26	Cysteinylglycine	45	Norcotinine
8	2-Oxoarginine	27	Cytidine monophosphate	46	Oxalacetic acid
9	21-Deoxycortisol	28	D-Cysteine	47	p-Cresol sulfate
10	3-Methylglutarylcarnitine	29	Dihydrogenistein	48	Palmitic acid
11	3-Sulfodeoxycholic acid	30	Ecgonine methyl ester	49	Palmitoylethanolamide
12	3b,17b-Dihydroxyetiocholane	31	Estrone	50	Phosphoenolpyruvic acid
13	4a-Carbinolamine tetrahydrobiopterin	32	Galactinol dihydrate	51	Pimelylcarnitine
14	5-Hydroxymethyluracil	33	Gamma-Glutamylcysteine	52	Pristanic acid
15	5,10-Methylene-THF	34	Glutarylcarnitine	53	Stearoylethanolamide
16	Acetyl-N-formyl-5-methoxykynurenamine	35	Guanosine monophosphate	54	Tantalum
17	Adrenic acid	36	Hexanoylcarnitine	55	Tetrahydrocortisone
18	Aldosterone	37	Hexanoylglycine	56	Thiophene
19	Alpha-Lactose	38	Hypotaurine	57	Vitamin A

Table S2. Metabo Analyst result pathway.

Pathway	Total	Expected	Hits	*p* value	Topology	PVal.Z	Topo.Z
Steroid hormone biosynthesis	137	1.0312	5	0.002803	0.12921	5.1953	1.3277
One carbon by folate	28	0.21076	2	0.018101	0.46667	3.4046	5.8511
D-Arginine and D-ornithine metabolism	5	0.037636	1	0.037102	0.2	2.7156	2.2766
Citrate cycle (TCA cycle)	50	0.37636	2	0.053155	0.20455	2.3705	2.3375
Pyruvate metabolism	64	0.48175	2	0.082191	0.17391	1.952	1.9269
Glutathione metabolism	75	0.56455	2	0.10775	0.13208	1.6921	1.3661
Taurine and hypotaurine metabolism	18	0.13549	1	0.12754	0.2	1.5302	2.2766
Glycolysis Gluconeogenesis	91	0.68498	2	0.14818	0.084746	1.3863	0.73166
Biosynthesis of unsaturated fatty acids	27	0.20324	1	0.18536	0.03125	1.1713	0.014569
Folate biosynthesis	32	0.24087	1	0.21589	0.057143	1.0249	0.36165
Fatty acid biosynthesis	49	0.36884	1	0.31177	0.022222	0.67216	−0.10644
Glyoxylate and dicarboxylate	53	0.39895	1	0.33265	0.08	0.60992	0.66804
Galactose metabolism	55	0.414	1	0.34286	0.061224	0.58089	0.41636
Alanine aspartate and glutamate	56	0.42153	1	0.34792	0.10204	0.56685	0.96349
Fatty acid elongation	57	0.42906	1	0.35293	0.013699	0.55311	−0.2207
Glycine serine threonine metabolism	68	0.51186	1	0.40572	0.047619	0.41929	0.23399
Tryptophan metabolism	80	0.60218	1	0.45864	0.0125	0.3016	−0.23677
Fatty acid metabolism	83	0.62476	1	0.47115	0.019417	0.27576	−0.14404
Retinol metabolism	83	0.62476	1	0.47115	0.25581	0.27576	3.0248
Pyrimidine metabolism	142	1.0689	1	0.66798	0.044248	−0.059355	0.1888
Purine metabolism	234	1.7614	1	0.84288	0.074713	−0.28263	0.59717
